# Development of handheld induction heaters for magnetic fluid hyperthermia applications and *in-vitro* evaluation on ovarian and prostate cancer cell lines

**DOI:** 10.1088/2057-1976/acbeaf

**Published:** 2023-03-10

**Authors:** Jorge L Castro-Torres, Janet Méndez, Madeline Torres-Lugo, Eduardo Juan

**Affiliations:** 1 Bioengineering Graduate Program, University of Puerto Rico, Mayagüez, Puerto Rico; 2 Chemical Engineering Department, University of Puerto Rico, Mayagüez, Puerto Rico; 3 Electrical and Computer Engineering Department, University of Puerto Rico, Mayagüez, Puerto Rico

**Keywords:** magnetic field generators, magnetic fluid hyperthermia, nanoparticles, laparoscopic and transrectal induction heaters

## Abstract

*Objective:* Magnetic fluid hyperthermia (MFH) is a still experimental technique found to have a potential application in the treatment of cancer. The method aims to reach around 41 °C–47 °C in the tumor site by exciting magnetic nanoparticles with an externally applied alternating magnetic field (AMF), where cell death is expected to occur. Applying AMFs with high spatial resolution is still a challenge. The AMFs from current and prospective MFH applicators cover relatively large areas; being not suitable for patients having metallic implants near the treatment area. Thus, there will be a clinical need for smaller magnetic field applicators. To this end, a laparoscopic induction heater (LIH) and a transrectal induction heater (TRIH) were developed. *Methods:* Miniature ‘pancake’ coils were wound and inserted into 3D printed enclosures. Ovarian (SKOV-3, A2780) and prostate (PC-3, LNCaP) cancer cell lines were used to evaluate the instruments’ capabilities in killing cancer cells *in vitro*, using Synomag^®^-D nanoparticles as the heat mediators. NIH3T3 normal cell lines were also used with both devices to observe if these cells tolerated the conditions applied. *Results:* Magnetic field intensities reached by the LIH and TRIH were 42.6 kA m^−1^ at 326 kHz and 26.3 kA m^−1^ at 303 kHz, respectively. Temperatures reached in the samples were 41 °C by the LIH and 43 °C by the TRIH. Both instruments successfully accomplished killing cancer cells, with minimal effects on normal cells. *Conclusion:* This work presents the first line of handheld medical induction heaters and have the potential to be a complement to existing cancer therapies. *Significance:* These instruments could enable the development of MFH modalities that will facilitate the clinical translation of this thermal treatment.

## 1. Introduction

Induction heating (IH) has a gamut of applications, owing to its efficient, safe, clean, and accurate method to deliver heat energy to a target object (metal or most electrically conductive materials). IH implementations vary from cooking in domestic appliances to heating magnetically susceptible materials inside a patient’s body [1–4]. A significant, but still mostly experimental IH application is magnetic fluid hyperthermia (MFH), which refers to the technique of accumulating magnetic nanoparticles (MNPs) in a tumorigenic tissue and exciting it with a high frequency alternating magnetic field (AMF) [5]. Here, the MNPs intrinsic heat dissipation activation raises the temperature in a localized manner up to 41 °C–47 °C, where cell death is expected to occur depending on the therapeutic conditions [6–10]. Over the years, there have been studies that evaluated the benefits of hyperthermia alone or in combination with chemotherapy, radiotherapy, immunotherapy, photodynamic therapy, and/or others [11–19]. These studies have proven that the combination of traditional, standard cancer therapies with hyperthermia modalities, results in increased damage to cancer cells. Other findings have evidenced that hyperthermia can indeed potentiate the effects of cytostatic drugs (e.g. bortezomib, 2-phenylethyenesulfonamide, cisplatin) [20–22], and even more by using low-intensity ultrasounds as intracellular delivery enhancers [23]. Efforts in the synthesis of iron oxide nanoparticles have also led to optimized MNPs that generate more heat during MFH experiments to achieve cell death [24].

Clinical translation of this technology has occurred at a slow pace, particularly because of several challenges that still need to be overcome, such as the ability to drive and accumulate MNPs uniformly in the target region via different delivery mechanisms, MNP heat dissipation rates *in vivo*, and the spatial resolution of magnetic field applicators. In Europe, however, this treatment is approved for clinical use in prostate adenocarcinoma and glioblastoma multiforme using the NanoTherm^®^ therapy [25, 26]. Recently, stage 2B clinical trials started in the USA for the focal ablation of prostate adenocarcinoma for cancerous lesions no larger than 2 cm^3^ [27]. Clinical trials for pancreatic cancer also started recently using the RCL NTT Generator and RCL-01 nanoformulation under the NoCanTher international project [28].

One of the challenges that MFH still needs to overcome is providing the treatment with high spatial resolution. It is implied that large magnetic field applicators such as the NanoActivator^®^ system [25] or medium magnetic field applicators such as the RCL NTT Generator [28] affect large regions of the patient. Although both devices provide a uniform magnetic field to the treatment area, their use is currently not suitable for patients having metal implants below their neck, although a recent report suggested revising the exclusion criteria [27, 29]. Larger constructs also limit how intense the magnetic field can be and its frequency, as applying rapidly changing magnetic fields could potentially generate unwanted eddy currents in the patient that could lead to non-specific heating of normal tissues. This product of the magnetic field intensity and the frequency, termed Atkinson-Brezovich exposure limit [30], could be exceeded if the therapy is localized to the target region. Devices that could ‘point and shoot’ without compromising healthy tissue could very well surpass this limit.

As for the tumors, some are challenging to treat, in part due to the inadequate delivery of the cytotoxic agent to tumorigenic region after the cytoreductive surgery and the poor drug penetration in tumors larger than 2.5 mm. Moreover, surgery becomes complicated when the tumor is invading blood vessels or other vital areas of the patient, limiting how much of the tumorigenic tissue can be removed [31]. Epithelial ovarian cancer, for example, requires an aggressive approach when trying to achieve maximal cytoreduction. This, in turn, leads to intra- and post-operative morbidity and mortality that has been found to also increase with age of the patient and the extensity of the tumor and surgery [32–34]. This is one of the reasons why the 5-year survival rate for ovarian cancer is low (49%), aside from the late detection of these silent killers [35]. Other malignancies, such as prostate cancer, typically present very high survival rates for local or regional stage lesions, in part, due to the extensive number of therapeutic approaches that currently exist [35]. This, however, is not without its faults. The treatments often impact the quality of life of men, especially when the nerve bundles at the sides of the prostate are damaged during the procedure or need to be removed, leading to psychosocial consequences [36–41]. Urinary and erectile dysfunction are some of the undesired side effects and the grade of severity varies depending on the therapy option and skill of the surgeon.

**Figure 1.**
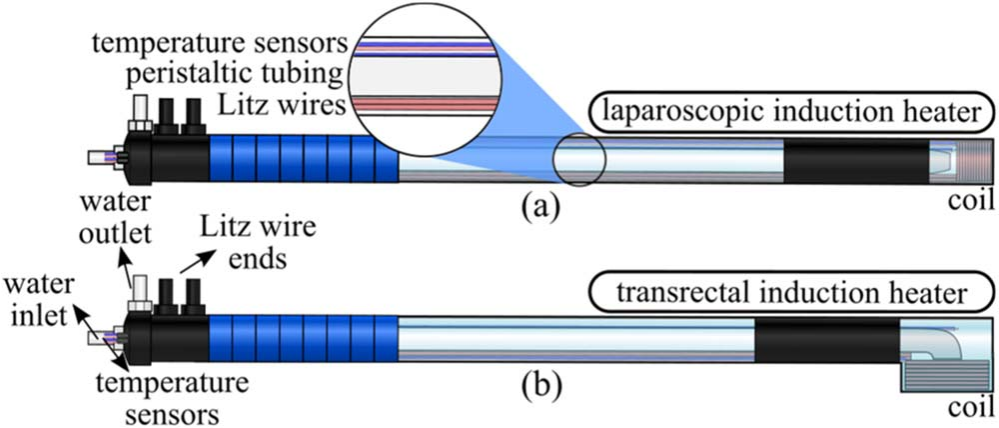
Conceptual diagram of the (a) laparoscopic induction heater and the (b) transrectal induction heater for magnetic fluid hyperthermia experiments. The Litz wire from the coil, peristaltic tubing, and temperature sensors are placed through the enclosures. Also, five input/output accesses are included for the peristaltic tubing, temperature sensors, water, and Litz wire ends. Once the peristaltic pump is turned ON, water starts filling the interior of the instrument through the water inlet/peristaltic tubing entrance, until it fills the device completely, and exits through the water outlet.

**Figure 2.**
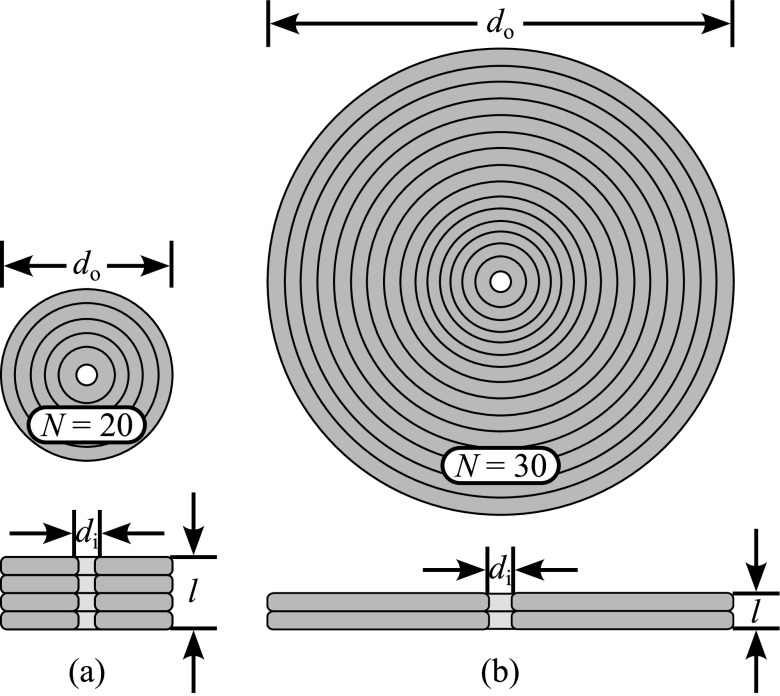
Laparoscopic and transrectal induction heater coils. (a) Laparoscopic induction heater coil and (b) Transrectal induction heater coil with the planar geometry and dimensions shown. Litz wire was used to build both coils.

Based on the previous discussion, there is a need for smaller magnetic field applicators. Highly localized approaches with high spatial resolution could potentially provide a solution to the challenges mentioned before by selectively killing cancerous tissue. To the best of the authors’ knowledge, there have been few reports on miniaturized magnetic field generators but designed to solely perform MFH-related studies in real-time using confocal microscopy [42, 43] or inverted microscopy *in vitro* or *in situ* [44], to evaluate drug release [45], or to gauge the heating performance of MNPs [46, 47]. Therefore, the purpose of this work was to design, construct, and evaluate two novel induction heating devices: (1) a laparoscopic induction heater (LIH; see figure 1(a)) and (2) a transrectal induction heater (TRIH; see figure 1(b)), as a first step towards their evaluation along with MNPs for the potential clinical translation of these types of nanoscale treatments.

The performance of the LIH was evaluated on A2780 and SKOV-3 cell lines, while the TRIH was evaluated on LNCaP and PC-3 cell lines. Both instruments were also evaluated on NIH3T3, to study how normal cells behave under MFH treatment conditions.

This work presents what is believed to be the first line of handheld medical induction heaters for cancer treatment. These innovative instruments could potentially enable the development of new MFH modalities that will certainly facilitate the clinical translation of this thermal therapy by providing treatment only where it is needed.

### 1.1. Overview

This work structures as follows: In section 2, the materials used and the methodology followed is thoroughly explained. Section 3 presents the results regarding both proposed devices and discusses the meaning behind them. Additionally, the limitations of this study are mentioned along with potential improvements and recommendations moving forward. Lastly, in section 4, the work is concluded with our last thoughts on the potential of our handheld induction heaters in the clinical setting.

## 2. Materials and methods

### 2.1. Magnetic field generator design

The advantage of a ‘pancake’ coil configuration for IH applications resides in the fact that some of the generated AMF appears on its surface. Therefore, it is suitable for applications that require surface heating and could have some degree of penetration depending on the intensity of the magnetic field. Other geometries, such as a helical configuration, require the target object to be inserted inside the coil [48–50]. A ‘pancake’ planar configuration met this project’s goal of applying AMFs to other surfaces. Hence, a 20-turn (for the LIH) and a 30-turn (for the TRIH) miniature multilayer ‘pancake’ coils were wound using a special high-frequency (200–350 kHz) wire known as Litz wire (MSW Wire Industries, Westlake Village, CA, USA). Litz wire is a conductor comprised of many twisted insulated strands that greatly minimizes the skin and proximity effects normally present in conductors at high frequencies by reducing the cross-sectional area of individual conductors [51]. It also counters the increase of the conductor impedance at higher frequencies, as the wire will maintain an AC resistance similar to its DC resistance. The wire used was composed of 120 strands of 42 AWG tightened with nylon, each one covered by a polyurethane insulation that allows it to withstand temperatures up to 155 °C.

The coils were wound counterclockwise on a nail (⌀ = 1.5 mm) that was inserted on a 10 cm × 10 cm slab of wood. Millimeter grid paper was glued to the wood to ensure the required coil diameter was met. Using this paper as a guide, twenty or thirty turns were made around the nail. Every three turns, Gorilla^®^ Super Glue (Gorilla Glue Company, Cincinnati, OH, USA) was poured on the windings to maintain the turns in place. The coils were then detached from the base with about 5 cm of unwounded wire. Finally, the ends of the coils were soldered to Litz wires of the same gauge on their respective devices. These wires were then soldered to 6 AWG Type 2 Litz wires (OSCO Ltd., Milton Keynes, UK) that run externally and connect directly to the electrical circuitry with set screw copper lugs. The devices were operated at resonance (see Supplementary Information; subsection A). For this particular design, resonance frequencies of 326 kHz and 303 kHz were selected for the LIH and TRIH, respectively. Other values of resonance frequencies (100–500 kHz) can also be obtained using different tuning parameters. Table 1 presents a list of all relevant parameters of the coils used for the LIH and the TRIH cases. See figure 2 for a depiction of the LIH and TRIH coils.

**Table 1.** Laparoscopic and transrectal induction heater coil parameters.

Parameters	Specifications (LIH coil)	Specifications (TRIH coil)	Units
Height (*l*)	6.39	2.98	mm
Outer diameter (*d*_o_)	10.53	29.50	mm
Inner diameter (*d*_i_)	1.57	1.30	mm
Turns (*N*)	20	30	unitless
Resistance (*R*_C_)	0.15	0.18	Ohms
Inductance (*L*_0._)	3.20	10.00	*μ*H

### 2.2. Magnetic field profile

To characterize the magnetic field generator, the proposed enclosure, which mimics known medical instruments, needed to be built (described in section 2.3). This housing, aside from being the body of the induction heater, acts as a cooling device. Without it, the coil would surpass the safe temperature limit (<155 °C) and be rendered unusable. The magnetic field profile of the coil used in both devices was measured with a custom-made magnetic field sensor (air coil configuration) [52], analogous to Connord *et al* (2016) [42]. The electrical current values were measured with a high-frequency Rogowski coil. These measurements helped predict the magnetic field intensities generated by the coil at a specific location/position and at a specific input current value. First, the magnetic field sensor was placed at the center of the coil surface using a positioner (David Kopf Instruments, Tujunga, CA, USA) and the Rogowski coil was put around one of the coil terminals. The input voltage of the wave generator was increased from 0 to 10 V, in increments of 0.5 V, to obtain the magnetic field intensity as a function of the input current. Then, the input voltage was set at the voltage level used for the MFH experiments (later discussed) and the sensor was moved upwards millimeter by millimeter to obtain the magnetic field intensity as a function of the axial distance. After obtaining these measurements, the positioner was positioned again at the surface of the coil and moved from end to end of the coil to obtain the magnetic field intensity as a function of the radial distance. Lastly, the last two steps were followed to obtain a better representation of the magnetic field intensity distribution. These values were acquired by moving the magnetic field sensor millimeter by millimeter from end to end of the coil and then increasing the axial distance by step of 1 mm. The process was repeated until the magnetic field intensity was negligible. The induced voltage values obtained from the magnetic field sensor and the Rogowski coil were later converted to magnetic field intensity and electrical current units using the equation (9) and equation (10) presented in Supplementary Information (subsection B) along with more information on the sensors and their calibration.

### 2.3. Cooling system design

With the idea of an MFH technique limited only to small tumor sizes, a special instrument was needed to deliver the heat in a minimally invasive manner. One potential end use of the LIH is during a laparoscopic procedure, a minimal invasive surgery where the surgeon inserts multiple tube-like instruments (with different functions) through small incisions in the patient’s body [53–56]. This instrument was designed with a tube-like structure, considering current laparoscopic instrument designs, and its performance is mainly limited by the magnetic generator parameters and the electrical current flowing through it. The TRIH was designed for prostate cancer malignancies. This instrument could be used to reach the prostate transrectally or to access the prostate by placing it in contact with the perineum. A normal prostate has a volume of approximately 25 cm^3^. Nevertheless, in cases of benign prostate hyperplasia (BPH) or prostate cancer, the prostate volume can increase to over 30 cm^3^. This was the rationale behind designing this coil larger than the one in the LIH.

**Figure 3.**
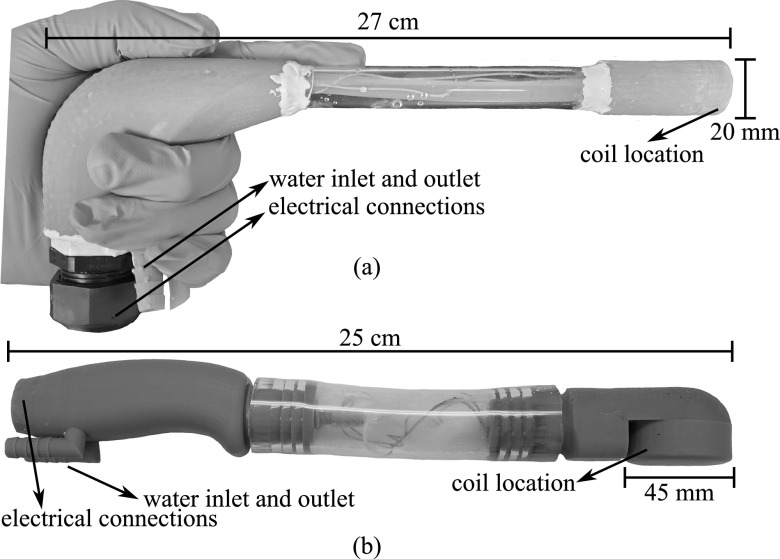
Laparoscopic and transrectal induction heater enclosures. (a) Laparoscopic induction heater and (b) Transrectal induction heater with lengths and diameter of the coil location shown. Both instruments were built with 3D printed parts and commercially available products.

The enclosures for each instrument were designed to have a medical device appearance, while also providing a means to maintain the internal temperature of the coil below 155 °C to avoid damage. Both embodiments were designed using NX software (Siemens, Plano, TX, USA) and were partially constructed by a Zortrax Inkspire 3D printer (Zortrax SA, Olsztyn, Poland) with epoxy-based resin from the same company. The constructs can regulate the coil temperature by circulating water (20 °C–25 °C) throughout the instrument similar as inside MFH coils [57]. The materials for the LIH design and its measurements were selected considering the dimensions of current laparoscopic instruments. A polycarbonate tubing (⌀_inner_ = 12.7 mm × ⌀_outer_ = 15.9 mm × 1.6 mm wall) (Small Parts Inc., Logansport, IN, USA) connected the 3D printed parts (handle and tip). The handle included the water inlet and outlets, as well as a nylon wet-location multi-cord grip (McMaster-Carr, Elmhurst, IL, USA) for the 6 AWG Type 2 Litz wires that connect directly to the circuit. The tip was where the coil was placed and served as a connection point for the Masterflex^®^ 25 L/S^®^ inner tubing (Cole-Palmer, Vernon Hills, IL, USA). This ensures that the water flows right into the coil before exiting the instrument. A cap at the tip ensured fast replacement of the coil if a problem was encountered. The overall length of the LIH was approximately 23 cm (see figure 3(a)).

The TRIH design was similar in the parts used, but different in shape. In this case, a tygon flexible tubing (⌀_inner_ = 25.4 mm × ⌀_outer_ = 31.8 mm × 3.2 mm wall) (McMaster-Carr, Elmhurst, IL, USA) connected the 3D printed parts (handle and tip). Similar to the LIH, the handle included the water inlet and outlets, as well as the nylon wet-location multi-cord grip for the 6 AWG Type 2 Litz wires that connect directly to the circuit. The tip in this case also enclosed the coil and served as a connection point for the 25 L/S^®^ inner tubing. This part had a 4-jet nozzle for improved heat removal of this larger coil. This design also had a cap for fast replacement of the coil if a problem was encountered. Because of the 3D printed enclosure, the instrument itself is slightly larger than what we aim to use in the future. However, this does not affect its performance, as the coil has the exact dimensions we designed for. The overall length of the TRIH was approximately 25 cm (see figure 3(b)).

### 2.4. Cell culture

Ovarian cancer cell lines A2780 and SKOV-3 were obtained from the institutional Cell Line Core Laboratory of the MD Anderson Cancer Center. Prostate cancer cell lines LNCaP and PC-3 were purchased from the American Type Culture Collection (ATCC, Manassas, VA, USA), as well as the NIH3T3 normal cell line. Ovarian cell lines were grown in RPMI-1640 (Sigma-Aldrich, St. Louis, MO, USA) supplemented with 15% fetal bovine serum (FBS; Life Technologies, Carlsbad, CA, USA), 2.0 g sodium bicarbonate, 0.1% gentamicin solution. LNCaP cells were cultured in RPMI-1640 supplemented with 10% FBS, 1.5 g sodium bicarbonate, 2.5 g glucose, 2.383 g HEPES, 0.11 g sodium pyruvate and 1% penicillin/streptomycin. The PC-3 cell line was grown in DMEM (high glucose) complemented with 10% FBS, 1.5 g sodium bicarbonate, 0.22 g sodium pyruvate and 1% penicillin/streptomycin. NIH3T3 cell line was cultured in DMEM (high glucose) supplemented with 10% FBS, 2.7 g sodium bicarbonate and 1% penicillin/streptomycin. All cell lines were incubated at 37 °C in 5% CO_2_. All the other reagents were purchased from Sigma-Aldrich. The cell lines were certified free of mycoplasma (read B/read A ratio below 0.9; see figure S7 in Supplementary Information) using the MycoAlert^®^ mycoplasma detection kit and MycoAlert^®^ assay control set (Lonza, Morristown, NJ, USA). Subsection C in Supplementary Information provides details regarding the management and cell counting of these cell lines.

### 2.5. Magnetic nanoparticles

As previously mentioned, MFH is a potential application of our instrument. Magnetic nanoparticles (MNPs) are required to dissipate enough heat to raise the temperature of the tumor up to 41 °C–47 °C, in presence of an AMF. Synomag^®^-D (Micromod, Rostock, Germany) MNPs with PEG 25000-OMe groups were used in this work (Lot # 06021104–01). These heat mediators are core–shell particles with a nanoflower maghemite core, covered by a dextran shell and functionalized with PEG-OMe. The MNPs are also suitable for MFH applications and have a prolonged circulation time in blood. Here, their magneto-physical properties are briefly described. The MNP hydrodynamic diameter was determined with the aid of dynamic light scattering (DLS) using the NanoBrook Omni particle size analyzer (Brookhaven Instruments Corporation, Long Island, NY, USA). Here, the MNPs were suspended in RPMI cell culture media from 0 to 72 h and measurements were taken every 24 h. The MNPs maintained their 70 nm hydrodynamic diameter size, meaning that they were not aggregating over time (see figure S8 in subsection D of the Supplementary Information). This was in accordance with the certificate of analysis from the company. The specific absorption rate (SAR) of the MNPs was 891.25 ± 21.66 W/g_Fe_ and was determined using the slope of the increasing temperature profiles of nanoparticles when exposed to the alternating magnetic field (28.33 kA m^−1^ at 258 kHz) of an EasyHeat 8310LI induction heater (Ambrell Corporation, Rochester, NY, USA). Iron quantification was conducted using an Infinite 200 Pro microplate reader (Tecan Group Ltd., Männedorf, Switzerland). The stock MNPs were 6.20 mg_Fe_ ml^−1^ when received and 5.99 mg_Fe_ ml^−1^ after filtration.

### 2.6. Heating capabilities at longer distances

One concern with magnetic fluid hyperthermia is how far can the magnetic field still heat nanoparticles up to a significant temperature (>40 °C). This is mainly because of unwanted heating on parts that are not the target or the opposite, not enough reach to activate the nanoparticles on the site. To provide more clarity on this matter, the heating capabilities of the larger device (TRIH) and the smaller device (LIH), were tested to demonstrate the temperatures reached when using various particle concentrations at different distances. This is particularly important for the TRIH since the potential end use would be on prostates accessed transrectally or in contact with the perineum, meaning that the magnetic field generated needs to reach the target. Four concentrations of nanoparticles ranging from 0.75 to 6 mg_Fe_ ml^−1^, with hydrodynamic diameter size of 70 nm, were used for this test. Five hundred microliters of each concentration were dispensed in the cavity of 35 mm cell imaging dishes (145 μm glass; Eppendorf, Enfield, CT, USA). Each sample was moved from 10 mm or 14 mm from the surface of the devices to 0 mm every 2 mm using a positioner. This was performed three times per concentration and every few runs a new sample was utilized to confirm that the temperature reached for that concentration at that particular distance was similar. Every 2 mm, a blank (water) was also subjected to the magnetic field to ensure the temperature rise of the samples was purely due to magnetic induction. The magnetic field intensity used was 42.6 kA m^−1^ and 26.3 kA m^−1^ at the planar coil surface of the LIH and the TRIH, respectively.

### 2.7. Magnetic fluid hyperthermia

**Figure 4.**
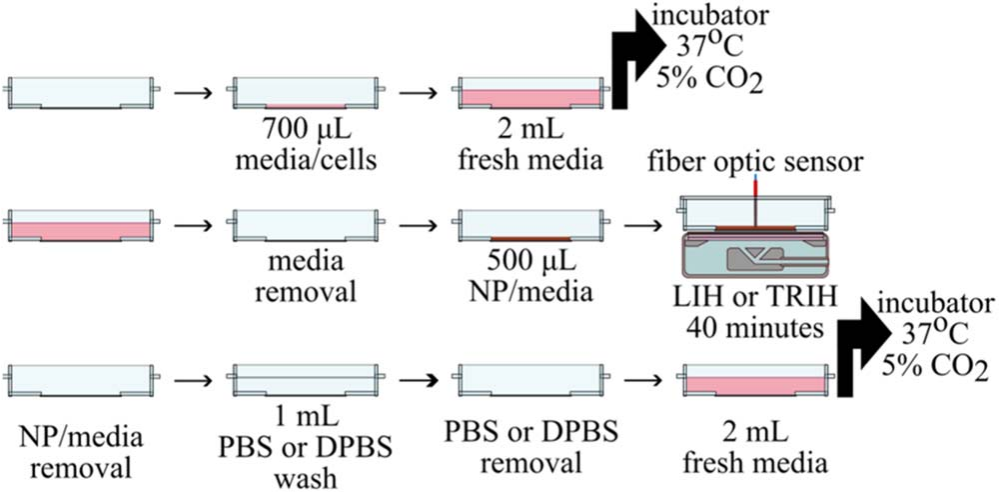
Graphical methodology of the plate management during the MFH experiments. Samples were on top of the devices for a maximum of 40 min of which 30 min were the treatment time at the desired hyperthermic temperature.

Cells were seeded on 35 mm cell imaging dishes (145 μm glass; Eppendorf, Enfield, CT, USA) at the seeding density required by each cell line (see table 2). Each plate was filled with 700 μl cell stock in the 18 mm cavity of the dish. The plates were left undisturbed in the incubator for 9–10 h to allow them to attach to the plate, except LNCaP cells, which had to be left undisturbed for 24 h. At this point, the plates were filled with 2 ml fresh media and returned to the incubator for 14 h (24 h for LNCaP) to complete the 24 h (48 h for LNCaP) before starting the experiment. The nanoparticle solution was prepared the day of the experiment (24 or 48 h after seeding). A volume of 2 ml was transferred from the nanoparticle stock (1.44 mg_Fe_ ml^−1^) to a centrifuge tube containing 2 ml of fresh media (2X) and FBS, yielding a nanoparticle solution at 0.72 mg_Fe_ ml^−1^. The order in which the plates were exposed to the magnetic field was obtained by a randomization tool. The process for each plate started by removing the culture media carefully at a specific region. Every pipetting step was done on the ‘S’ or south position of the plate. After media removal, the plate was filled with 500 μl of the nanoparticle solution or fresh media. After each run, the nanoparticle solution or the culture media was removed to wash the plate one time with buffer. Then, the buffer was discarded to fill the plate with 2.0 ml of fresh media. The dish was then moved to the incubator for 24 h or 48 h before detaching and suspending the cells to count them manually and assess cell viability with the Trypan Blue exclusion assay (see subsection C of the Supplementary Information) [23, 58, 59]. See figure 4 for a graphical depiction of the plate management. Each experiment consisted in three samples per condition and was repeated two other days for a total of nine samples per condition.

**Table 2.** Information on cell lines used and culture details.

Cell Line	Cells per Plate	Passage	Medium	Buffer
A2780	76,000	27–32	RPMI + 15%FBS	PBS
SKOV-3	25,000	27–31	RPMI + 15%FBS	PBS
LNCaP	60,000	15–19	RPMI + 10%FBS	DPBS
PC-3	25,000	10–14	DMEM + 10%FBS	DPBS/EDTA
NIH3T3	25,000	34–37 (LIH) 40–42 (TRIH)	DMEM + 10%FBS	PBS

LIH: Experimental samples (E) were exposed to an AMF of approximately 42.6 kA m^−1^ at 326 kHz for a maximum of 40 min. The treatment time was set to 30 min after reaching around 40.5 °C to maintain the temperature around 41 °C. This usually happened between 8–10 min. The AMF negative control (A) group was also subjected to the same conditions as the experimental sample. While these groups were being exposed to the AMF, two control groups were left in the incubator; one with only cells (C) and another group with cells and MNPs (N) for 40 min. This last group was used solely to ensure that the MNP concentration employed in the experimental group was not harmful (or contaminated) to the cells, at least, during the treatment time.

TRIH: Experimental samples (E) were exposed to an AMF of about 26.3 kA m^−1^ at 303 kHz for a maximum of 40 min. The treatment time was set to 30 min after reaching around 41.5 °C in 5–8 min to maintain the temperature around 43 °C. Everything else was conducted in the same manner as described above.

### 2.8. Experimental setup

A 20 MHz 33220A function/arbitrary waveform generator (Agilent Technologies Inc., Santa Clara, CA, USA) was used to drive the coils. Amplification of this input signal was performed using a 1000 W RF power amplifier, model 1140LA (Electronics & Innovation Ltd., Rochester, NY, USA). The amplified signal runs through the L-matching circuit that enables maximum power transfer from the source to load (coil). Since the coil dissipates heat due to resistive losses, a Masterflex^®^ peristaltic pump circulates water through the instrument’s body to remove excess heat. A simple air-cooled heat exchanger configuration maintains the circulating water at a constant temperature of about 24 °C to avoid the temperature increasing throughout the duration of the experiment. Temperatures of the test samples (e.g. cells, MNPs) and surroundings were monitored with a FOTEMP-PLUS signal conditioner using TS3–10 mm-06 fiber optic temperature sensors (Optocon AG, Dresden, Germany) and stored in a computer using the FOTEMP-ASSISTANT 2.3 configuration and data logging software, also from Optocon AG. The temperature of the samples was measured by passing the fiber optic thermal sensor through a lid with a hole and the surrounding temperature was measured by placing another fiber optic sensor close to the plate. The reusable lid was cleaned with ethanol after each run to avoid contamination. Subsection E in Supplementary Information shows top-view and side-view of the samples placed on the devices (see figure S9 and figure S10), as well as an example of the temperatures reached during MFH (see figures S11 and S12).

### 2.9. Statistical analysis

Bars represent the mean of a total of *n* = 9 replicates for each condition from three independent experiments. Error bars represent the standard error from three independent experiments. All the plots were generated using OriginPro 2021 software from OriginLab Corporation^®^ (Northampton, MA). Comparisons were performed using the Student’s t-test two-tailed distribution, two-sample with unequal variance) in Minitab^®^ statistical software. A *p*-value below 0.05 was considered statistically significant.

## 3. Results and discussion

This section presents the magnetic field profiles of the proposed devices, as well as their performance as potential MFH based therapies for malignancies needing highly localized AMFs. A discussion on the results concludes this section.

### 3.1. Magnetic field profile

The magnetic field profile of each device was acquired with a series of magnetic field intensity and electrical current measurements.

**Figure 5.**
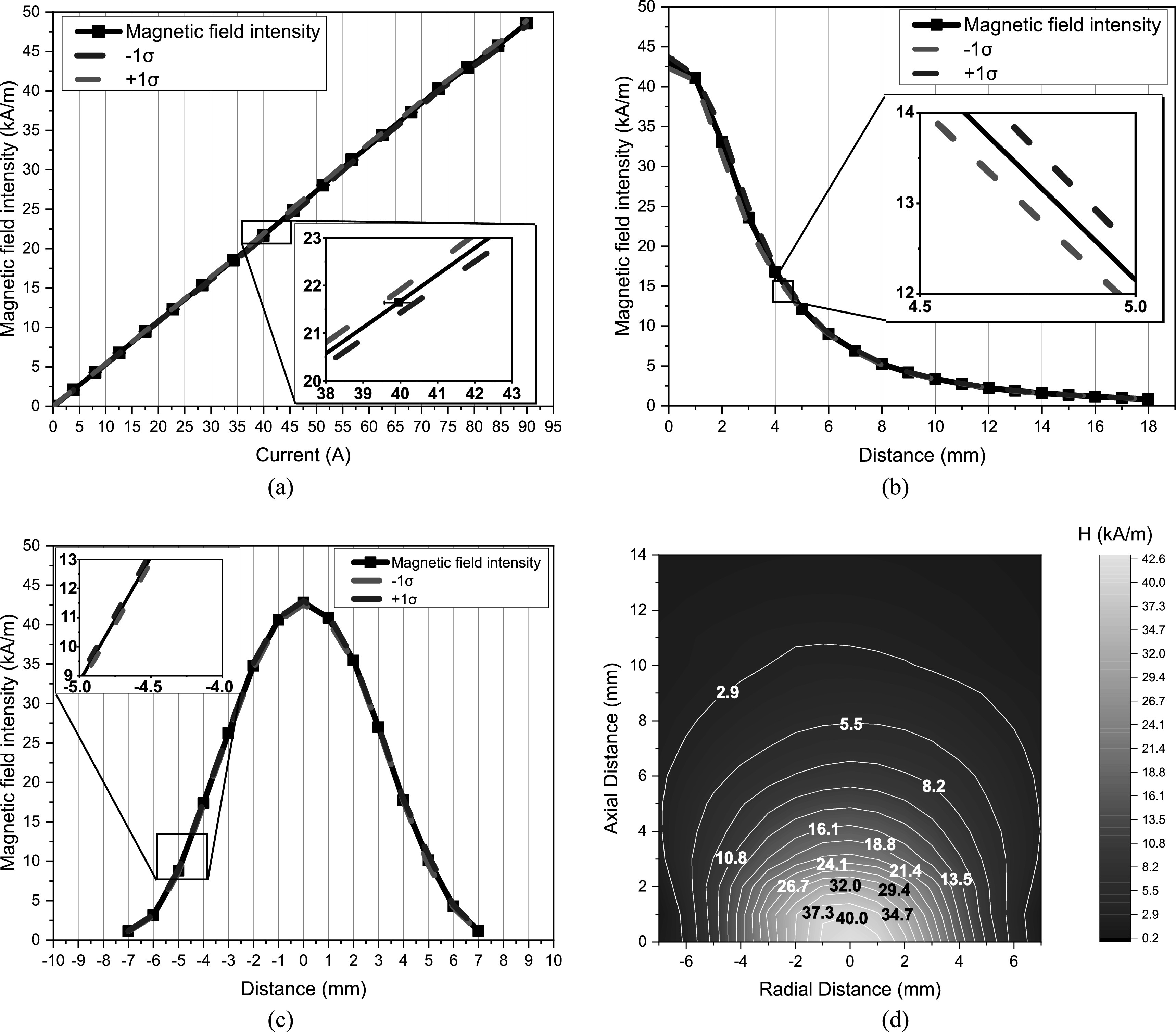
Magnetic field profile of the laparoscopic induction heater obtained experimentally. (a) Magnetic field intensity at the central part of the surface of the instrument as a function of the current through the coil. (b) Magnetic field intensity as a function of axial distance from the surface of the coil. (c) Magnetic field intensity as a function of radial distance across the surface of the device. Each point is the mean of three measurements whereas red and blue dashed curves represent a standard deviation from the mean. (d) Color map representing a cross-sectional slice of the magnetic field intensity distribution.

**Figure 6.**
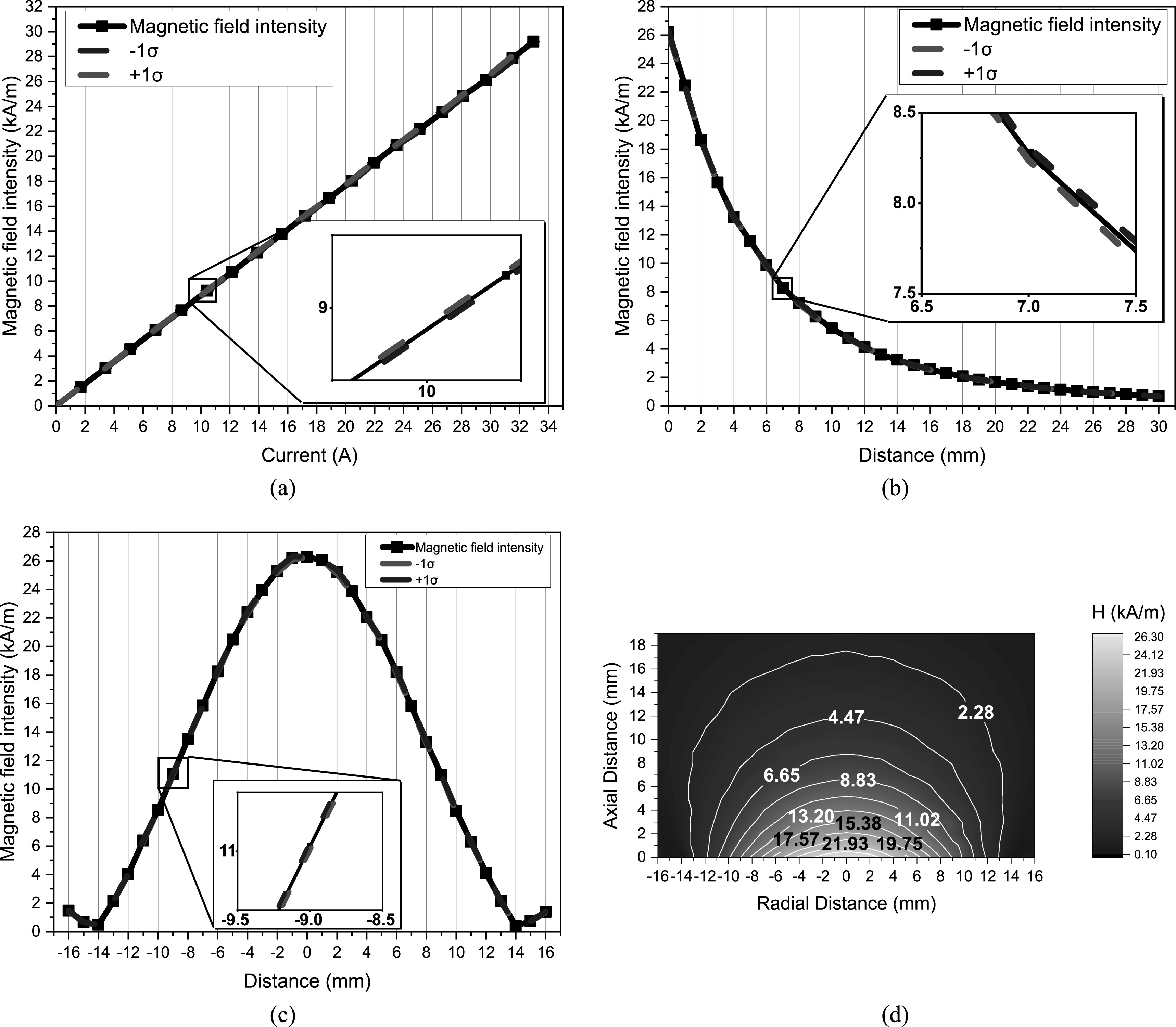
Magnetic field profile of the transrectal induction heater obtained experimentally. (a) Magnetic field intensity at the central part of the surface of the instrument as a function of the current through the coil. (b) Magnetic field intensity as a function of axial distance from the surface of the coil. (c) Magnetic field intensity as a function of radial distance across the surface of the device. Each point is the mean of three measurements whereas red and blue dashed curves represent a standard deviation from the mean. (d) Color map representing a cross-sectional slice of the magnetic field intensity distribution.

Figures 5 and 6 illustrate the magnetic field profile of the LIH and TRIH instruments, respectively. The values for both set of plots were obtained experimentally as described in section 2.2. This provides information on what magnetic field intensities are to be expected when increasing the input current. Figures 5(a) and 6(a) show the linearity of the magnetic field, at the surface of the instrument as a function of the current through the coil. The maximum coil current value was 90 A for the LIH and 33 A for the TRIH. This resulted in a magnetic field intensity value of 47 kA m^−1^ for the LIH and 29 kA m^−1^ for the TRIH, which were the last values in the plots. Figures 5(b) and 6(b) depict the exponential behavior of the magnetic field intensity as a function of the axial distance from the center of the coil. Here, the magnetic field intensity was set to the conditions used for the proposed MFH experiments, which was 42.6 kA m^−1^ and 26.3 kA m^−1^ for the LIH and TRIH, respectively. It is of note here that the magnetic field decreases exponentially with increasing axial distance. Figures 5(c) and 6(c) display the magnetic field distribution along the radial axis at the surface of the instrument using the same magnetic field intensities mentioned before. In this case, the plots show the behavior of the magnetic field as the observer is moved from end to end of the coil. The magnetic field intensity will always be stronger at the center. Finally, figures 5(d) and 6(d) exhibit the color map representing a cross-sectional slice of the magnetic field intensity distribution.

### 3.2. Heating capabilities at longer distances

**Figure 7.**
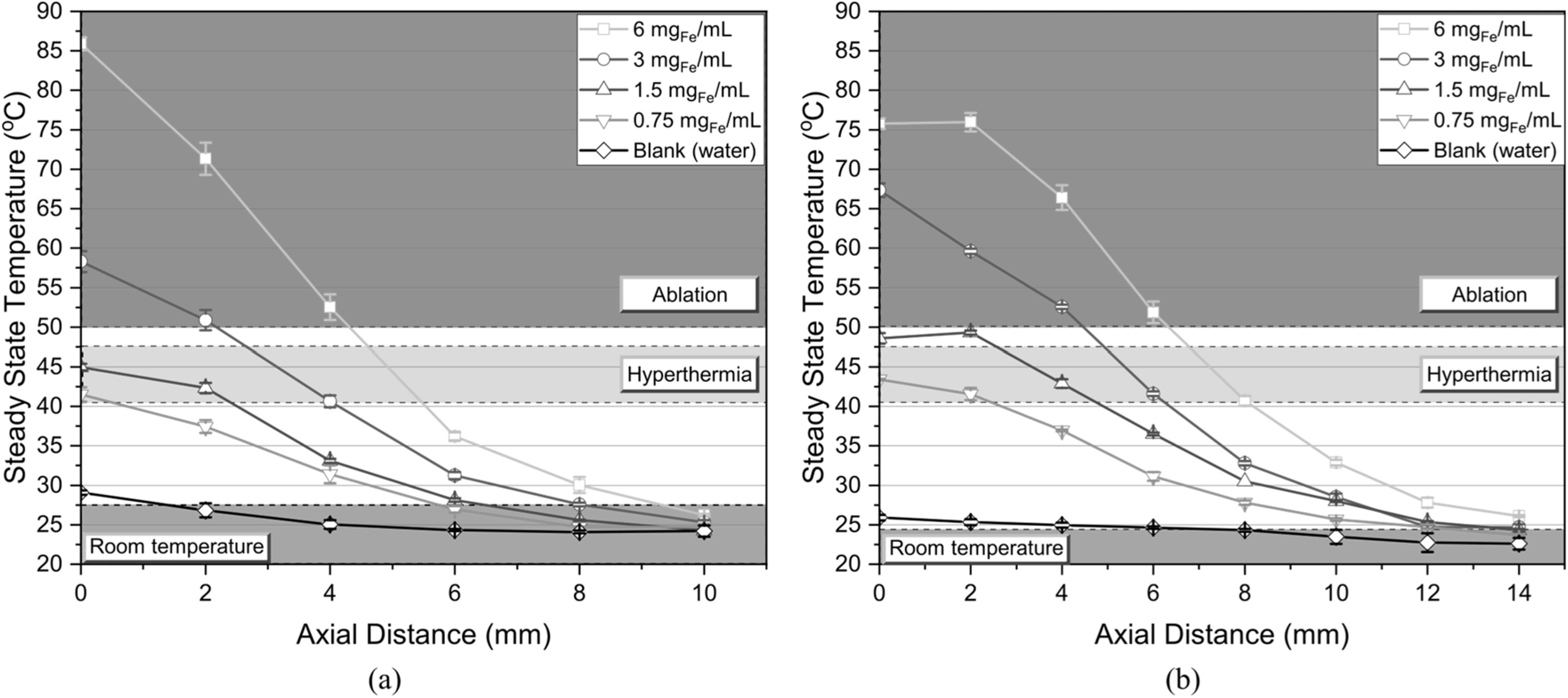
Steady-state temperatures for each MNP concentration at increasing distances from the (a) LIH and (b) TRIH coil surfaces. The runs were made every 2 mm until the temperature raise was not meaningful (<30 °C). The plot shows steady-state temperatures for MNP concentrations of 6 mg_Fe_ ml^−1^ (yellow squares), 3 mg_Fe_ ml^−1^ (blue circles), 1.5 mg_Fe_ ml^−1^ (red upward triangles) and 0.75 mg_Fe_ ml^−1^ (green downward triangles). A blank was included for each condition, represented by the black diamonds curve. Each point represents the mean of three samples.

The LIH and the TRIH coils were used to determine the heating capabilities of the proposed devices at longer distances. Various MNP concentrations were used to determine the extent of the magnetic field and observe at what distances the MNPs could still reach relevant hyperthermic or even ablative temperatures. Figure 7 illustrates the steady state temperatures reached by each MNP concentration at increasing distances from the surface of the devices. The runs were made every 2 mm until the temperature raise was not meaningful (<30 °C). A blank was included for each condition, symbolized by the black diamonds curve. Each point represents the mean of three samples. Relevant hyperthermic levels shown in the green region of the plots can still be reached at increasing distances by increasing the MNP concentration. Low concentrations (0.75 mg_Fe_ ml^−1^) reached approximately 41 °C and 43 °C at the surface of the LIH and the TRIH, respectively. The LIH could maintain hyperthermic temperatures between 41 °C and 47 °C at longer distances by increasing the MNP concentration. No hyperthermic temperatures were observed after 6 mm.

The TRIH could still heat the 0.75 mg_Fe_ ml^−1^ concentration up to 42 °C at a 2 mm distance due to the larger size of the coil compared to the LIH. A temperature of about 41 °C could still be reached at 8 mm by the TRIH if a concentration of 6 mg_Fe_ ml^−1^ was used. Notice that the temperatures reached from 0–2 mm (in figure 6(b)) are similar, except the 3.0 mg_Fe_ ml^−1^ concentration, confirming that the strongest magnetic fields are in that region, as shown in figures 6(c) and 6(d). Reaching hyperthermic levels would also be feasible at longer distances with a stronger AMF. No significant temperature rise was seen after 10 mm for the LIH and after 14 mm for the TRIH, as these samples were very close to room temperature.

It is of note that by miniaturizing the induction heating system and offering portability and highly localized magnetic fields, among other features, the LIH and the TRIH currently have a penetration depth limitation (<1 cm). It can also be argued that malignancies with more than 2 mm of depth would not be exposed to the same magnetic field intensities, but injecting MNPs at different concentrations depending on the distance could compensate for this to accomplish uniform heating of the target region.

### 3.3. Magnetic fluid hyperthermia

The first potential application of the LIH was thought to be on intraperitoneal malignancies, such as ovarian cancer, and which are regarded as silent killers. The TRIH was designed for prostate cancer after feedback (as part of the NSF I-Corps^TM^ Site and Teams programs) from several surgical oncologists from Puerto Rico and USA. Although these novel instruments could have several applications in the medical realm as well as outside of it, it was decided to focus them first on these malignancies.

**Figure 8.**
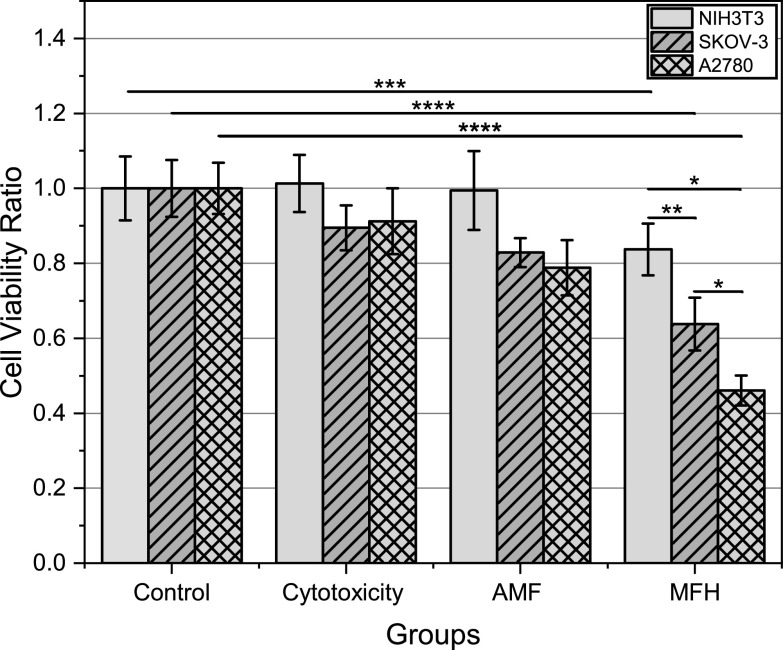
Laparoscopic induction heater results. Cell viability ratio of the control, cytotoxicity, magnetic field control (AMF) and experimental (MFH) groups obtained using Trypan Blue assay by manual counter. Each error bar represents the standard error of nine samples from three independent experiments per cell line (NIH3T3, turquoise plain bars; SKOV-3, orange diagonal pattern; A2780, gray diamond pattern). Extremely significant, *p* < 0.0001; highly significant, *p* < 0.001; very significant, *p* < 0.01; significant, *p* < 0.05; not significant, *p* > 0.05.

**Figure 9.**
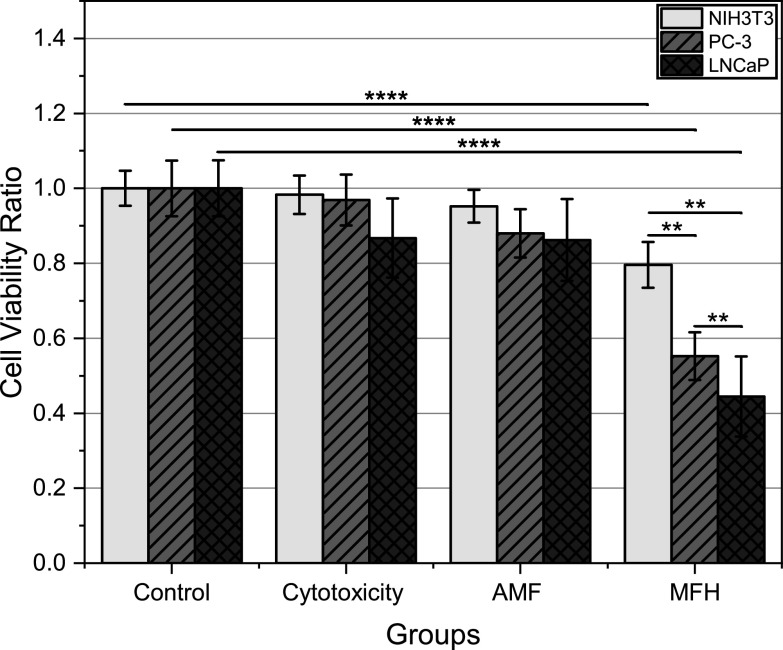
Transrectal induction heater results. Cell viability ratio of the control, cytotoxicity, magnetic field control (AMF) and experimental (MFH) groups obtained using Trypan Blue assay by manual counter. Each error bar represents the standard error of nine samples from three independent experiments per cell line (NIH3T3, turquoise plain bars; PC-3, violet diagonal pattern; LNCaP, blue diamond pattern). Extremely significant, *p* < 0.0001; highly significant, *p* < 0.001; very significant, *p* < 0.01; significant, *p* < 0.05; not significant, *p* > 0.05.

Both proposed handheld induction heaters were then evaluated on cancerous and normal cell lines to determine their capabilities in killing cancer cells and how well normal cells could withstand MFH conditions. Figures 8 and 9 illustrate cell viability ratios for each condition tested per cell line. Each bar represents the mean of nine replicates from three independent experiments. Cytotoxicity, AMF and MFH groups were normalized against the control to obtain these values.

The LIH was tested on A2780 and SKOV-3 ovarian cancer cell lines and on the NIH3T3 normal cell line. Figure 8 shows the results from the MFH experiments on these targets. The cell lines tolerated the MNP dose used, at least throughout the duration of the experiment. This was evidenced by the viability ratios of the cytotoxicity group. Both cancer cell lines present a mean reduction down to about 90%, whereas NIH3T3 is close to 100% viability, hinting that healthy cells could endure the dose used if MNPs end up in their vicinity. Cells without MNPs were exposed to the AMF generated by the device to ensure it could damage cancer cells purely by the action of the AMF on the MNPs. While a reduction down to about 80% was observed for A2780 and SKOV-3 cells, the normal cell line maintained around 100% viability. Values below 90% for cancer cells for this condition were unexpected. Although the exact cause is unknown, it can be hypothesized that this reduction could be because the product of the magnetic field intensity and frequency produced by the LIH (13.89 × 10^9^ Am^−1^Hz) surpasses the exposure limit established by Atkinson-Brezovich (4.85 × 10^8^ Am^−1^Hz). This would still be safe for a patient due to the highly localized approach that this work presents, contrary to larger systems if they were using these intensities and frequencies. Cancer cells could have lower tolerance to this product than healthy cells, but more studies would need to be performed before drawing conclusions. Finally, there was a significant reduction in viability for both cancer cells under MFH conditions when compared against the other groups. Even though the normal cell line also presented a significant lower value in viability (83.7%), it was still significantly higher than those displayed by the cancer cells. Both cancer cell values were also significantly different from each other, as expected. SKOV-3 cells and A2780 cells have been reported to have differences in hyperthermia sensitivity, due to the connective tissue growth factor (CTGF) preventing energy-stressed cell death after hyperthermia [60]. In their study, Hatakeyama *et al* (2016) classified cells with LT50 (lethal temperature 50 °C) above the median, SKOV-3, as hyperthermia resistant cells and below the median, A2780, as hyperthermia sensitive cells [60]. This is one of the possible reasons why SKOV-3 cells presented a reduction down to only 63.8%, whereas A2780 cells obtained 46.1% viability. Even though temperatures reached by the MFH group average 41 °C (mild hyperthermia), the results still align well with those reported by Court *et al* (2017) [21] and Mérida *et al* (2020) [23] with a temperature of 43 °C (moderate hyperthermia) using larger systems. For specific significance values between groups and conditions, see Supplementary Information (subsection E; table 4).

The TRIH was evaluated on LNCaP and PC-3 prostate cancer cell lines and on the NIH3T3 normal cell line (see figure 9). These cell lines also tolerated the utilized MNP dose, at least throughout the duration of the experiment. PC-3 cells displayed a higher tolerance to the MNP concentration used than LNCaP cells, as they present a viability close to 100% along with the NIH3T3 cells. Higher viability ratios can be observed for the AMF group than those reported on figure 8. Again, this could potentially be due to the Atkinson-Brezovich exposure limit, as the product of magnetic field intensity and frequency for the TRIH (7.97 × 10^9^ Am^−1^Hz) is lower than for the LIH. More studies need to be conducted on this matter. Finally, a significant reduction in viability can be observed for both cancer cells under MFH conditions when compared against the other groups. The normal cells also presented a lower viability (80%), but still significantly higher than both cancer cells, similar to what was observed on figure 8. PC-3 and LNCaP viability ratios were also significantly different from each other. Though there have been numerous articles on these cell lines and combination studies using radiation and/or chemotherapeutic drugs along with hyperthermia, none of the studies used MNPs as the heat mediator *in vitro* [61–69]. Instead, they employed different methods to reach hyperthermic levels, such as using an incubator or hot water [70, 71]. One article reported results of MFH on the LNCaP cell line, but the temperature reached by the samples was not stated [72]. Another study showed reduction in viability of LNCaP cells also using MFH, but under low frequency conditions (44 Hz) and did not disclose the final temperature of the samples [73]. To the best of the authors’ knowledge there have been no other reports on MFH and both of these cancer cell lines *in vitro*, being this the first article on the matter. Therefore, the results shown in this work could not be directly compared with other reports at present. Despite the lack of MFH studies on LNCaP and PC-3, reports on hyperthermia using other methods on these cell lines can at least provide information on the resulting cell viability after treatment. Zhang *et al* (2016) reported a reduction in viability down to about 80% for PC-3 and about 60% for LNCaP when subjected to 43 °C for 1 h in an incubator [70]. Another article presented approximately the same remaining viabilities, but interchanged [71], and was in accordance with the suggestion from Sahin *et al* (2011) that PC-3 cells were slightly more thermosensitive than LNCaP cells [74]. In our study, these experiments were conducted at an average temperature of 43 °C for 30 min. Our viability values were even lower than the first two studies. This can be attributed to the fact that MFH can reduce viability to a greater extent than water-based or incubation-based hyperthermia [10, 20, 75]. Even though we obtained a higher viability for PC-3 cells (55.2%) than for LNCaP cells (44.5%), values such as these are encouraging for future *in vivo* studies. For specific significance values between groups and conditions, see Supplementary Information (subsection E; table 5).

Figures 8 and 9 show promising results to continue investigating the performance of the devices on tumors and/or animal models and distinct nanoformulations tailored to the application. There are still many hurdles ahead of this technology. However, many researchers around the world are still making new discoveries on MNPs and their interaction with the tumor microenvironment, hyperthermia treatment/prediction plans, and many technologies to achieve cell death. In the future, a toolbox can be provided to clinicians for any malignancy size in any location within the body. The instruments presented in this article aim to become the complement to other existing therapies that cannot possibly treat all lesions using the same method and potentiate others that have already proven their worth against cancer.

### 3.4. Limitations

The maximum value of magnetic field intensity is mainly limited by the capacity of the power amplifier. The magnetic field intensity may be increased by using a power amplifier with higher wattage (>1000 W) but must go in hand with a more efficient heat removal approach to prevent overheating of the coil. Small-sized coils also limit the magnetic field intensity. However, we had to keep the size of the coils relatively small due to the highly localized approach that this work pursues. In this case, we traded off the size of the coil for portability, focused magnetic fields and potential low cost.

There are several coil geometries/configurations for many applications. We selected the ‘pancake’ coil configuration over other designs because of its use in surface heating applications. Although its generated magnetic field is not completely uniform, other geometries require the target object to be inside the coil. This would be impractical in the clinical setting unless the magnetic field applicators were made larger such as the approved system in Europe. However, the drawbacks of this method were discussed in section 1 of this manuscript.

The capabilities of the LIH and the TRIH in heating MNP suspensions at longer distances were tested. While the penetration depth in the case of the TRIH is not yet large enough to cover a whole prostate, it is enough to treat the region closest to the rectum. This limitation could be resolved by applying MNPs at various concentrations that would depend on the distance between the instrument and the target, as discussed section 3.2.

Additional work is required to further explore the capabilities of these instruments on samples that better represent the microenvironment of the tumor (e.g. animal tissues or animal models). This future work would be useful to determine the penetration depth of each device and the temperature reached in this type of setting, as other factors (e.g. tumor vascularity, blood perfusion) affect the MNP distribution and heat dissipation. Regardless, the results presented on this paper pave the way for more challenging studies in the future.

## 4. Conclusion

This paper has presented two innovative highly localized MFH approaches by miniaturizing the working coil and maintaining relatively high magnetic field intensities. At the expense of magnetic field uniformity, our systems offer portability, highly localized magnetic fields, and potential low cost. Both devices were also tested on appropriate cancer and normal cell lines as a first step, yielding quantitative evidence on their capabilities in killing cancer cells. MFH results indicated marked differences between normal and cancer cells and between the latter, showing that both devices can damage cancer cells with minimal damage to normal ones. Both proposed instruments are believed to be the first line of handheld medical induction heaters for cancer treatment. Both devices have the potential to be a complement to existing, larger size magnetic field generators, for therapies that require more precision or for patients having metallic implants near the treatment area. For example, a type of therapy alongside the standard of medical care; surgery followed by concomitant chemotherapy/radiotherapy and MFH. If successful, this innovation could improve clinical outcomes and quality of life of the patient. These novel instruments could also potentially enable the development of new MFH modalities that will certainly facilitate the clinical translation of these types of nanoscale treatments.

## Data Availability

All data that support the findings of this study are included within the article (and any supplementary files).
